# Resistance of IoT Sensors against DDoS Attack in Smart Home Environment

**DOI:** 10.3390/s20185298

**Published:** 2020-09-16

**Authors:** Ladislav Huraj, Marek Šimon, Tibor Horák

**Affiliations:** 1 Department of Applied Informatics, University of SS. Cyril and Methodius, 91701 Trnava, Slovakia; marek.simon@ucm.sk; 2 Institute of Applied Informatics, Automation and Mechatronics, Faculty of Materials Science and Technology in Trnava, Slovak University of Technology in Bratislava, 91724 Trnava, Slovakia; tibor.horak@stuba.sk

**Keywords:** Internet of Things, DDoS, smart home, security, IoT sensors, personal assistants

## Abstract

Smart devices along with sensors are gaining in popularity with the promise of making life easier for the owner. As the number of sensors in an Internet of Things (IoT) system grows, a question arises as to whether the transmission between the sensors and the IoT devices is reliable and whether the user receives alerts correctly and in a timely manner. Increased deployment of IoT devices with sensors increases possible safety risks. It is IoT devices that are often misused to create Distributed Denial of Service (DDoS) attacks, which is due to the weak security of IoT devices against misuse. The article looks at the issue from the opposite point of view, when the target of a DDoS attack are IoT devices in a smart home environment. The article examines how IoT devices and the entire smart home will behave if they become victims of a DDoS attack aimed at the smart home from the outside. The question of security was asked in terms of whether a legitimate user can continue to control and receive information from IoT sensors, which is available during normal operation of the smart home. The case study was done both from the point of view of the attack on the central units managing the IoT sensors directly, as well as on the smart-home personal assistant systems, with which the user can control the IoT sensors. The article presents experimental results for individual attacks performed in the case study and demonstrates the resistance of real IoT sensors against DDoS attack. The main novelty of the article is that the implementation of a personal assistant into the smart home environment increases the resistance of the user’s communication with the sensors. This study is a pilot testing the selected sensor sample to show behavior of smart home under DDoS attack.

## 1. Introduction

Internet of Things (IoT) devices are becoming common in everyday life. Using a variety of sensors, they routinely provide and collect a variety of household information. In addition, their ubiquitous connectivity, as well as their ability to communicate with each other or to trigger action on the event due to the sensed data from the sensors, increase their popularity. Although IoT devices simplify and automate everyday tasks, they also bring security vulnerabilities. Because the security measures used to protect IoT devices are still not sufficient, IoT devices are becoming a possible point through which an attacker can penetrate and attack the smart home environment infrastructure. Attacks can then cause various effects such as damage to hardware, disruption of system availability, cause system outages, and even physically harm individuals.

The vulnerability of IoT devices is mainly based on their limited computing power, their limited resources (memory, radio bandwidth, and battery source), insufficient updating of IoT devices by the manufacturer and heterogeneity of IoT devices in terms of hardware, software, and communication protocols as well as the amount of data exchange involved. These facts make it difficult to deploy robust security mechanisms and allow attackers to disable or compromise IoT devices, and often to disable or compromise the entire environment using these IoT devices. The main principles of security, which are confidentiality, integrity, and availability (CIA), are also important when implementing IoT devices [1]. IoT manufacturers place little emphasis on the safety of IoT devices due to financial savings. An example is, e.g., updating the firmware of IoT devices, where the update is often impossible or enormously difficult for the end user.

In addition, smart home networks are also more vulnerable to security threats due to the heterogeneous nature of the devices connected to them. They also have some special vulnerabilities because nodes in a smart home network are usually located in both heterogeneous and hosted environments [2].

The concept of smart home environment is also built on IoT devices, where the effort is to integrate home security and automation into one unit. IoT devices connected to the Internet and collecting data from their sensors allow the homeowner to remotely monitor and real-time control their home. The consumer market offers a huge number of IoT devices monitoring the home, such as light spots, lightbulbs, smart thermostats, motion sensors, smart locks, fire alarms, security cameras, even vacuum cleaners or robot lawnmowers. The advent of low-cost smart home environments has been made possible by falling prices for IoT sensors and other hardware, as well as the advent of cloud technologies [3].

New phenomena are smart-home personal assistant systems such as Google Home Assistant or Amazon Alexa. Smart-home personal assistant system is a smartphone-based natural language input device. Such assistant system can monitor and control home IoT devices of multiple providers through a cloud-based smart home platform. Different sets of connected IoT devices are used in different households. In addition to voice interaction with the user, assistants are able to interact with other IoT devices in the smart home environment and perform tasks such as compiling task lists or setting alarms. To control home Internet of Things devices, the user can use the smart-home personal assistant application directly, or even the speech interface, although the current technology for direct voice control of home Internet of Things devices is not yet sufficient for the entire ecosystem of the Internet of Things. Therefore, specialized voice activation technology and users’ preferred keywords are used to allow users to more naturally customize their speech communication with their devices [4].

The role of smart-home personal assistant systems is to collect, analyze, and use the data of their users. As with IoT devices, security and privacy needs to be taken into account. Nevertheless, these systems also have a number of privacy and security issues, and their design flaws cause unforeseen problems in everyday life. Even errors in their basic mechanisms threaten users’ privacy and device security, and their unusual behavior increases confidence issues. Introducing transparency in the data collection behavior of these systems could lead not only to resolving the issue of trust, but also to better controlling security and privacy [5].

The fact that the smart home environment is connected and available in real time is convenient for the user. On the other hand, in a smart home environment, the real space is endangered, as well as the privacy of people in the smart home environment is compromised, and an attacker can gain access to personal information that is collected about people by sensors installed in IoT devices. Because the smart home environment is connected to the Internet, attacks on it can occur from anywhere in the world. An attacker can monitor the lives of people in the house, or personal information may be used in an unauthorized way. In addition to the threats associated with smart home Internet connections, other threats associated with other technologies used by IoT devices such as radio frequency identification (RFID), wireless sensor network (WSN), Wi-Fi, 3G, 4G, or 5G are possible [6].

Losing control of IoT systems in a smart home environment can pose a high security risk, as a large number of IoT applications are used in routine life; the risk factor is higher compared to the traditional network [7]. Loss of control of the system can cause various unpleasant or dangerous situations such as failure to turn on or off the light, jamming of doors, inability to change the temperature in the room, or even worse such as a fire outbreak when a fire detector does not report a fire, or the possibility of intrusion into the house by criminals when the sensor movements do not report movement in the house. It is unsatisfactory if the user cannot rely on receiving a notification from sensors in the smart home environment in a critical situation, or if he is unable to control the IoT device using the IoT application.

One of the possible attacks that an attacker can perform against the victim’s device over the Internet is a Distributed Denial of Service (DDoS) attack. During a DDoS attack, an enormous number of requests are sent from a number of computers from different locations. The purpose of a DDoS attack is not to get unauthorized access or to obtain sensitive data; the DDoS attack is only intended to flood the victim’s device, and thus prevent access to the requested service [8]. It is IoT devices that are often misused to create DDoS attacks, which is due to the weak security of IoT devices against misuse. The article looks at the issue from the opposite point of view, when the target of DDoS attack are IoT devices in a smart home environment. The article examines how IoT devices and the whole smart home will behave if they become victims of a DDoS attack conducted on the smart home from outside. From the point of view of DDoS attacks performed in this case study and described in the article below, the availability of services provided by the smart home environment, on which DDoS attacks have an impact, is critical.

There are many types of attacks on the smart home environment and they are based on the individual parts of which the smart home environment consists. Heartfield et al. in [9] presents smart home attack vector classification criteria, where categories of IoT platforms with threats relating to the communication medium, control software used, threats in the supply chain, side channel attacks, and the sensory channel are described. In addition, they present 24 different smart home cyber threats against the taxonomy criteria to identify shared characteristics between threats that help to identify key areas for developing defenses. Habibzadeh et al. in [10] summarizes the major privacy and security aspects of smart homes where the challenges include DDoS attacks, large scale and stealth attacks, and the domains involve privacy leakage, device-level security, and system management.

DDoS attacks against IoT devices can disrupt their services as well as their Internet connection. DDoS attacks can significantly increase the operating costs of smart homes in terms of bandwidth and device life; damage the confidence of the general public in the continued adoption of IoT devices; and possibly cause a loss of revenue and damage the market. There is a significant need to understand the impact of DDoS attacks on smart home devices [11].

Given the DDoS attack used in this article, it is necessary to give a real example of DDoS attack from the life of a smart home with unpleasant consequences. Such is, for example, the targeted DDoS attack on two smart buildings in Lappeenranta, Finland, in November 2016, with the interruption of heating systems during the Finnish winter. Most of the controlled automatic systems, such as heat distribution, ventilation, and hot water, tried to automatically prevent the attack. As a result, the system got into an endless loop, restarted every few minutes, and denied remote administrator access to the devices. The heating systems were inoperative for more than a week. Such attacks on civilian infrastructure can weaken the comfort of the population as well as endanger people’s safety [12,13].

Researchers at the institute for critical infrastructure technology [14] state that much more serious and destructive attacks can be expected. The Lappeenranta attack on the smart home environment seems to be just a test run that tested the capabilities of the botnet, probed the defense, or planned an attack on a larger target.

Another example of the need to test the vulnerability of smart environment communications with sensors is a centrally controlled vehicle handling sensor system, where the Internet of Vehicles (IoV) is even more complex than a smart home. The environment contains sensors such as internal sensors and actuators (accelerator, steering wheel, and brakes), external sensors (cameras, GPS, and lidars), internal cockpit sensors (alertness, health sensors, and tone of voice) or messages (crowd-sourced info and tweets), and alarms reports on the vehicle state, etc. There is also a DDoS attack and loss of communication with sensors a threat, with DDoS attacks by hackers and malicious agents conducted as V2V communication [15].

Most current research in this area is focused on DDoS attacks and IoT in terms of involving IoT devices in DDoS attacks, e.g., [16,17,18]; less attention is paid to DDoS attacks conducted on the IoT devices themselves, e.g., [2,19], nor on the response of IoT sensors or the possibility that communication with the human user has not been taken into account. The possibility of disabling the smart-home personal assistant system using a DDoS attack is addressed in, e.g., [20], where Overstreet et al. present an attack using a DDoS SYN flood attack on Amazon Echo version 2, testing the verbal response to the questions asked. Tushir et al. in [11] report DDoS attacks on most popular IoT devices in the smart home environment, and also analyze their impact on the power consumption of affected devices. They did not deal with the response of IoT sensors to this attack.

DDoS attacks with an impact on the user’s communication with the sensors have not yet been reported in the literature. This is the main novelty of this article, as well as the finding that the implementation of a personal assistant into the smart home environment increases the resistance of the user’s communication with the sensors.

The remainder of this paper is organized as follows. Section 2 briefly explains the main methods applied in the case study involving DDoS attack, device profiles, and testbed environment. Scenarios of the proposed experiments as well as the experimental results are described in Section 3. Next, the results are discussed in Section 4 and the conclusion and future work is stated in Section 5.

## 2. Materials and Methods

**Figure 1:**
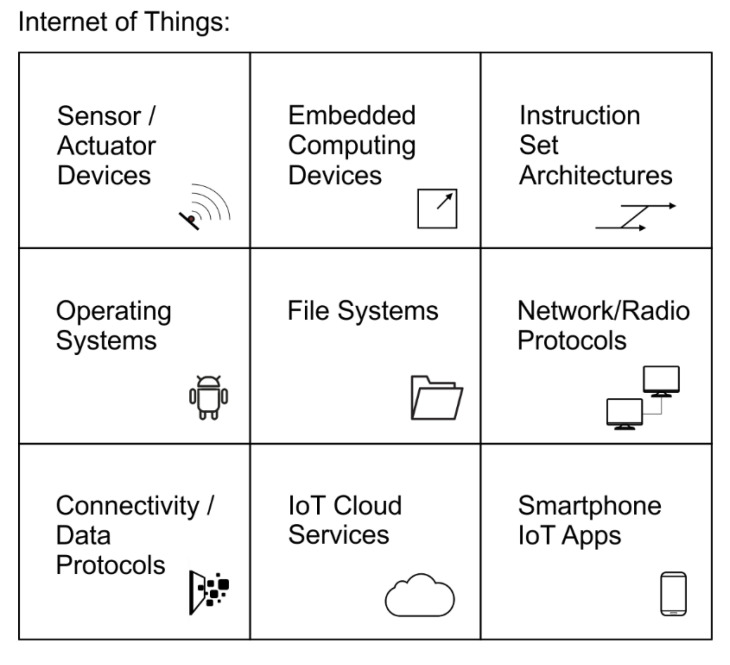
Various components of Internet of Things.

Internet of Things (IoT) can be simply referred to as an internetwork, where smart physical things can communicate with each other and with virtual objects via the Internet. Smart home is an application of IoT technology. The goal of the smart home environment is the integration of home automation and security so that the homeowner can also remotely control and monitor the environment [1,3,4]. As mentioned above, IoT devices have limited resources, resulting in their low computing power and low storage space. All IoT-specific components need to be considered for IoT security. Figure 1 shows the individual components of the Internet of Things, namely, Sensor/Actuator Devices, Embedded Computing Devices, Instruction Set Architectures, Operating Systems, File Systems, Network/Radio Protocols, Connectivity/Data Protocols, IoT Cloud Services, Smartphone IoT Apps.

The case study in this article focuses only on one area of components, namely sensors. Sensors are one of the characteristic features of IoT that sense the physical environment. Examples of IoT sensors are motion, fire, proximity, temperature, magnetic, humidity, air quality, power, light, water flow, color and more [21].

Communication with sensors takes place via special protocols such as Zigbee, Z-Wave, Thread Bluetooth Low Energy, EnOcean, etc. The ZigBee and Z-Wave protocols are used for communication with IoT sensors in the case study, where an intermediary node is required to connect the sensors to the Internet, e.g., hub, router, bridge, or other gateways. The task of the intermediary node is to connect other IoT sensors to the created network. The IoT sensor itself cannot route any communication or authorize other devices to connect to the network and can only communicate within the network through its intermediary node. The intermediary node is constantly in operation and continuously powered, while the end IoT sensor can go into low power mode to save energy. On the other hand, the Thread protocol does not need an intermediary node to connect to the Internet, but it provides itself an connectivity to any IP-based network, involving Wi-Fi, Ethernet, and the Internet in general [22]. Attacks on the security of the protocols used for IoT sensors are a real problem. Attacks on the ZigBee protocol have already been shown, e.g., at Black Hat 2015 [21].

The experiments in this article focus only on the Internet connection; i.e., use attacks conducted over the Internet and are not aimed at violating Network/Radio protocols defining communication standards between smart home devices. Any complex sensor can be simplified to the communication point at the level of communication (whether using Internet protocols TCP/IP or specific protocols such as Zigbee, Z-Wave, etc.). The sensors in the case study were used in this way.

In general, security attacks can be classified into two main categories. The first category are passive attacks. In passive attacks, an attacker attempts to obtain information about the system without affecting system resources. A passive attack obtains information about the system through monitoring and eavesdropping on the system and transmitted messages, without modifying them. These types of attacks are difficult to detect because they do not change the data, but only gather something from the data. The second category of attack are active attacks. In active attacks, the enemy uses the information that was collected during the passive attack and uses it to try to change system resources or change system operations. Active attacks include, e.g., modification of information or attempting to gain unauthorized access to system resources. Common examples of active attacks are worms, malware, ramsomware, password cracking, or denial of service [23].

In the case of IoT devices, the following types of attacks can be considered in particular [24]:

1. Physical attack—attack interferes with the hardware components.
2. Side channels attack—attack uses any encryption device to achieve information from various side channels.
3. Cryptanalysis attack—attack tries to break cipher text to obtain a plain text or a key to retrieve information of a device.
4. Software attack—various security measures are impacted in this attack in an IoT device.
5. Network attack—attack based on broadcast nature of various communication channels.

The attacks carried out in the case study are network attacks. These are DDoS attacks that try to overwhelm the victim with an enormous number of requests and cause the system to malfunction, thus preventing the legitimate use of a service. Commonly, IoT devices are exploited on DDoS attacks, where an attacker uses a network of compromised IoT devices to send a lot of traffic in order to flood and overwhelm the target, resulting in inability of the victim’s services. The experiments performed in the case study, on the other hand, place the IoT device in the position of a victim in a smart home environment in order to disable the user’s communication and control of the IoT sensors of these devices.

Ali et al. in [23] list the basic security requirements in the smart home environment: User and Device Authentication, Monitor the Network, Integrity, Availability, and Confidentiality. Of these requirements, Availability is most at risk in a smart home due to a DDoS attack, as the Availability requirement ensures that all network services and resources are available in a protected form at all times.

### 2.1. DDoS

Cyber attacks are a chronic matter of the Internet in today’s world. Any attack that aims to disrupt the smooth running of a service in any way is a Denial of Service (DoS) attack. If such an attack involves several machines attacking at the same time (usually tens of thousands), the attack is called distributed, in short DDoS.

The target of a DDoS attack is a specific machine device, service, or even the infrastructure of the institution. A successful attack disrupts the normal operation of a service or network and causes damage to the owner, which is quantifiable according to the nature of the system. There are different ways to implement a DDoS attack. Next, the basic types of DDoS attacks used in the case study are described. The ability of users to use IoT sensors during such attacks was tested. DDoS attack performance was examined in real time according to various scenarios.

#### 2.1.1. SYN Flood Attack

**Figure 2:**
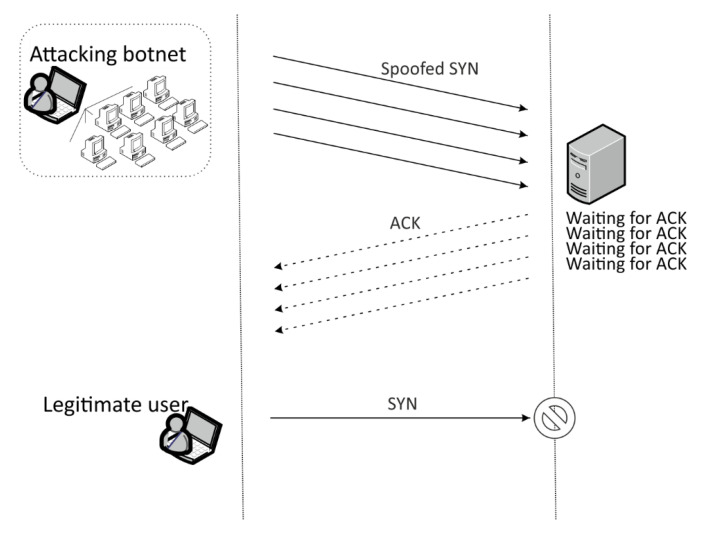
SYN flood Distributed Denial of Service (DDoS) attack.

During TCP connection establishment in classic server–client model, server has to receive a SYN (synchronize) packet from a client. Consequently, the server binds some resources for such half-open TCP connection and sends back a SYN-ACK packet to the client. Since the server resources are limited, if a client never sends back an ACK (acknowledgment) packet and if a large amount of SYN packets from many other malicious clients are sent, the resources available on the server can be exceeded and the server cannot connect to any other new clients, Figure 2. Such form of DDoS attack is called SYN flood [25].

#### 2.1.2. HTTP Get Flood Attack

**Figure 3:**
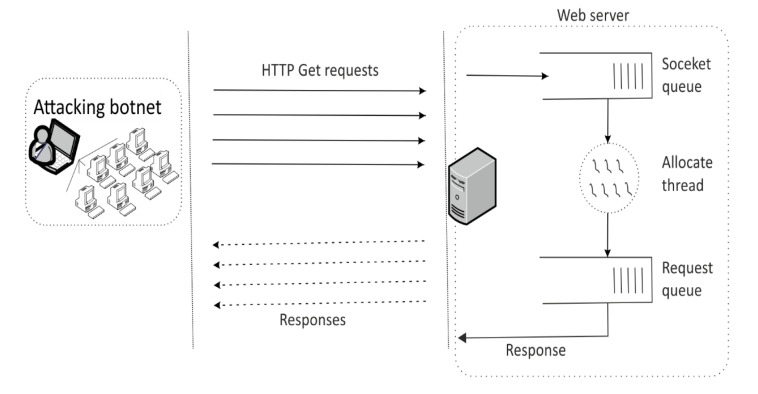
HTTP Get flood DDoS attack.

HTTP Get flood attack is one of the most usual types of DDoS attacks of an application layer. During the HTTP Get flood attack, an attacker uses legitimate IP addresses which appear to be authentic sources, so the web server receives and processes the HTTP Get requests continuously. If a large number of requests are sent, the web server is overwhelmed and the server cannot process any other new HTTP Get requests, Figure 3. A detection scheme based on identifying spoofed IP addresses or a blacklisting of IP addresses is not fully successful [26].

#### 2.1.3. SSL/TLS Flood

**Figure 4:**
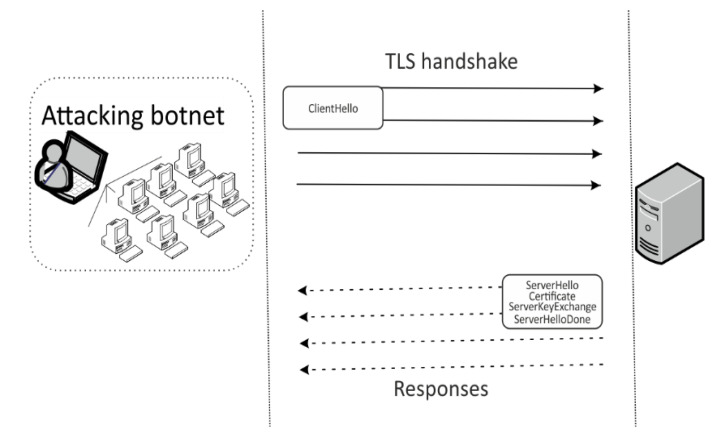
SSL/TLS flood DDoS attack.

SSL/TLS flood or SSL/TLS DDoS attack uses the need to expend computing power of the server when building a secure TLS connection. The attacker loads the server’s resources beyond its limits and shuts it down during TLS negotiation by sending a large amount of garbage to the server or constantly asking to renegotiate the connection. SSL/TLS flood mainly consumes web server’s CPU resources, Figure 4. Mitigating an attack is not easy because establishing an SSL/TLS connection requires a lot of resources and the attacker’s requests appear to be legitimate [27].

### 2.2. Ansible Real-Time Attack Environment for DDoS Experimentation

The article uses a test environment based on the Ansible orchestration tool to implement DDoS attacks. Ansible real-time attack environment uses thousands of DDoS attack nodes of various types to ensure better experimentation in order to get as close as possible to the scale of the “real world” with the greatest possible scientific integrity. Moreover, Ansible orchestration allows easy manipulation by system administrators in a distributed heterogeneous environment. Ansible tool provides automated configuration, coordination and management of computer systems and software.

Ansible manages a group of remote computers from a central node. Remote computers do not require the installation of any additional software and are connected to the central node via the SSH protocol. Ansible executes and removes modules on machines using only Python and a few other packages.

In addition, the Ansible structure works on a client–server topology, which logically follows the structure of the botnet network used in DDoS attacks. Using Ansible makes it easy to create a test environment on heterogeneous devices on a network. Previous use of this tool for DDoS attacks has demonstrated the ability of Ansible real-time attack environment to perform scientific experiments aimed at testing systems under DDoS attacks [28].

**Figure 5:**
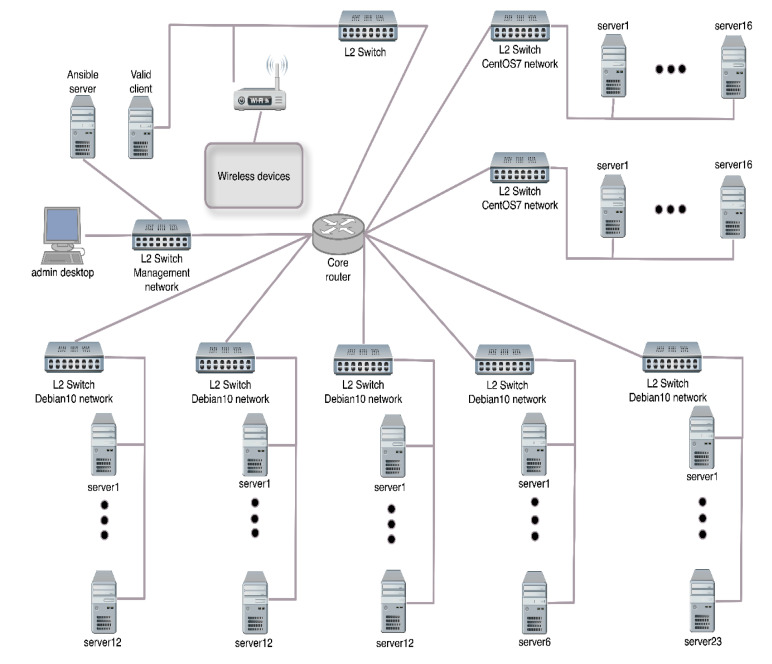
Structure of Ansible real-time attack environment for DDoS experimentation.

To describe the Ansible real-time attack environment used in the case study in detail, the Figure 5 illustrates the structure of the attack environment. The attack environment involved 82 physical or virtual servers. The number of physical servers was 14 running OS Debian10, 2 GB RAM, Intel (R) Core (TM) 2 Quad CPU Q8400 @ 2.66 GHz. Virtual servers were also used. Each server was based on Kernel-based Virtual Machine (KVM, www.linux-kvm.org) module that allows the kernel to function as a hypervisor. Specifically: 36 servers running Debian10, 1 GB RAM, Intel (R) Xeon (R) CPU E3-1230 v3 @ 3.30 GHz, and 32 servers running CentOS7, 1 GB RAM, one core Intel (R) Xeon (R) CPU E5-2620 v4 @ 2.10 GHz. Up to one hundred virtual nodes were emulated by each server, increasing the number of unique IPs on the network. Then the final number exceeded the value of 8000 unique IPs attacking the target. The bandwidth of all links in the network topology was 1 Gbps. It should be noted that the structure of the attacking botnet can be easily changed through the Ansible tool by adding additional machines.

HTTP Get flood attacks were implemented through a benchmarking tool for HTTP server ApacheBench; SYN flood attacks via a Python script and an open source packet manipulation tool Scapy.

### 2.3. Examined Sensors

This section describes individual IoT devices as well as IoT sensors used to test the impact of a DDoS attack on the smart home environment.

#### 2.3.1. Fibaro

Fibaro is a global brand operating in the Internet of Things sector, which provides solutions in the field of home automation systems. The company offers a number of sensors and actuators such as motion, light, temperature, vibration sensors, smoke, flood and motion detectors, door, and window opening sensors or smart electrical sockets and smart thermostatic radiator heads.

In addition to smart sensors, the company also focuses on a smart home management unit, which wirelessly controls connected sensors and controllers and is able to add any sensor with a binary output to the system. Furthermore, components such as a switch in an electrical socket with a function for measuring energy consumption, blinds, and shutters, a lighting controller or relay switches can be used. The system is able to work with a home weather station, wireless speakers, or cameras. The Fibaro devices use Z-Wave protocol.

**Figure 6:**
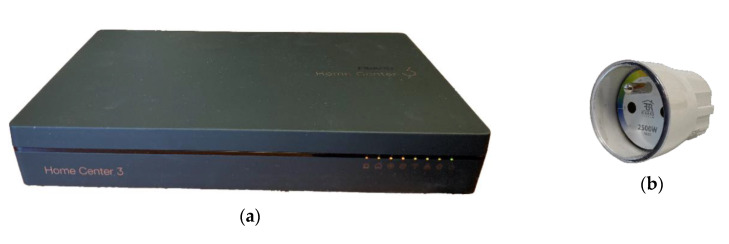
Fibaro system: (**a**) Gateway for Fibaro system management—Home Center 3; (**b**) Fibaro Wall Plug.

For the purposes of the case study, the brand new Fibaro system management unit—Home Center 3, Figure 6a was used. The control of the Fibaro system in three ways was used during the study: (i) with Fibaro Home Center mobile app provided by the manufacturer, (ii) with local web interface provided by the manufacturer, and (iii) control with external mobile app of Homey Athom or of voice personal assistants Amazon Alexa and Google Assistant.

As a representative sample of IoT sensor based on Z-Wave protocol, a Fibaro Wall Plug was used, Figure 6b. Fibaro Wall Plug is used to measure the energy consumption of electrical appliances. Each plug has a unique identification number, allowing to connect more than one plug to a closed area and communicate with each one separately. The Fibaro Wall Plug uses short-range Z-Wave technology to exchange information and communicates through the Fibaro Home Center. The Fibaro Wall Plug measures the energy consumption of connected electrical appliances and reports it via the Z-Wave. It also turns on or off the power to devices connected to this plug according to the instructions [29].

Our previous experience with the Fibaro Home Center Lite small gateway and the Honeywell thermostat already points to the fact that it is possible to use a DDoS attack to disable a specific IoT device during the attack and prevent communication between the smart home user and the IoT device, thus preventing communication and collecting information from the IoT sensor. The Honeywell thermostat was unable to send an alarm notification to the mobile application and so failed to inform in time the smart home user of the threats detected by the sensor during the attack [30].

#### 2.3.2. Hue Philips

At least one smart bulb and one bridge, also referred to as a hub or gate, are needed to create a connected lighting system. The bridge provides communication with the smart bulb via the ZigBee protocol and subsequently communicates via the Internet. The bridge uses an RF transmitter to send commands to the lights and a receiver to receive the light’s status. The bridge can manage several different lights in different rooms in the house.

**Figure 7:**
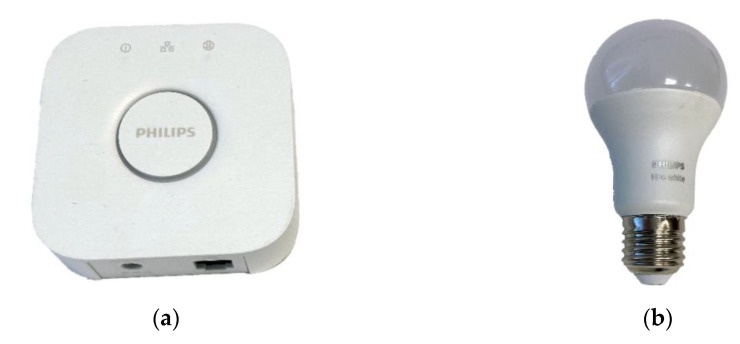
Philips Hue lighting system: (**a**) Philips Hue Bridge 2.0; (**b**) Smart LED bulbs Philips Hue White 9W E27 model LWB010.

For the purposes of the case study, Philips Hue Bridge 2.0 was used, which acts as the gateway between the Internet/LAN and the lights, Figure 7a. During the study, control of the connected bulb was used in two ways: (i) with Philips Hue mobile app provided by the manufacturer, and (ii) control with external mobile app of Homey Athom or of voice assistants Amazon Alexa and Google Assistant.

As a representative sample of IoT sensor based on ZigBee protocol a Smart LED bulb Philips Hue White 9W E27 model LWB010 was used, Figure 7b. A user can turn on or off as well as to change brightness of the light. The Hue Bridge provides full control of the lights. The user can turn off the bulbs by regular light switch, which, however, has the effect that off-state bulbs cannot be controlled by mobile applications anymore. Smart LED bulb consists of (i) RF receiver which allows the bulb to communicate with the controller; (ii) processing unit which is responsible to process and execute the commands received. For instance, when increasing the brightness, the processing unit increases the brightness incrementally over a short period to avoid sharp light changes that most people find unpleasant; (iii) Drivers and LEDs. The brightness level of the bulb is determined by the signal received by the driver that turns the LED on and off at a very fast rate. The brightness is determined by the ON duty cycle [31].

### 2.4. Smart-Home Controllers and Smart-Home Personal Assistant Systems

Another type of technology used in a smart home environment are smart-home controllers and smart-home personal assistant systems. This type of technology is able to connect to various other smart devices and through them, to the IoT sensors, as well as to smartphones and tablets.

The role of the smart-home controller is to solve the problem of technological fragmentation which arises in the implementation of the ecosystem of smart homes. Different IoT devices use different communication protocols, which can be confusing for smart home users. One solution would be to create a universal protocol that would be used by all devices in a smart home environment. However, such an approach seems unrealistic, as devices are highly heterogeneous with different functionality, and it is difficult to design an all-in-one protocol for each device from different manufacturers. Another, simpler solution is to implement a gateway that supports many protocols, which act as a central controller for all smart devices in the home. Such a gateway is a smart-home controller. However, even in this case, manufacturer’s list compatibility, as there is a huge number of different IoT devices on the market and it is not easy to support each of them [32]. In the case study, the smart-home controller Athom Homey is used, and it is also possible in this category to include also the above-mentioned Fibaro system.

On the other hand, the goal of smart-home personal assistant systems is to eliminate the process of typing. So that the user does not have to type a query into the Internet browser, the dictation method is used here, where the user enters the query as a voice request. Based on speech recognition, the Smart-home personal assistant converts the request into text, performs the required action, and announces the result again by voice. The systems use artificial intelligence techniques such as speech recognition, natural language processing, dialogue systems and speech synthesis [33]. These include, for example, the Amazon Alexa, Google Assistant, Apple Siri, or Microsoft Cortana.

With the advent of smart-home personal assistant systems, users have acquired a number of new intelligent services. They aim to provide a wide range of services by consistently collecting various kinds of information such as personal profiles, location or time. While the IoT concept dates back to the 20th century, the connection to personal assistant systems was not available until about 2014 with the Amazon Echo release and the integration of IoT devices into the agent’s affordances. The interconnection of personal assistant systems and the IoT field bonds the area of virtual agents with their interaction with physical objects and users, together with networked physical devices, including sensors [34]. The personal assistant system is intended to serve as a user interface for connecting to a smart-home and to pick up and interpret voice requests to utilize other devices and services, either using scheduled actions or voice orders. Managing smart home devices through a smart-home personal assistant system obviously requires connecting the smart-home personal assistant system to the device.

**Figure 8:**
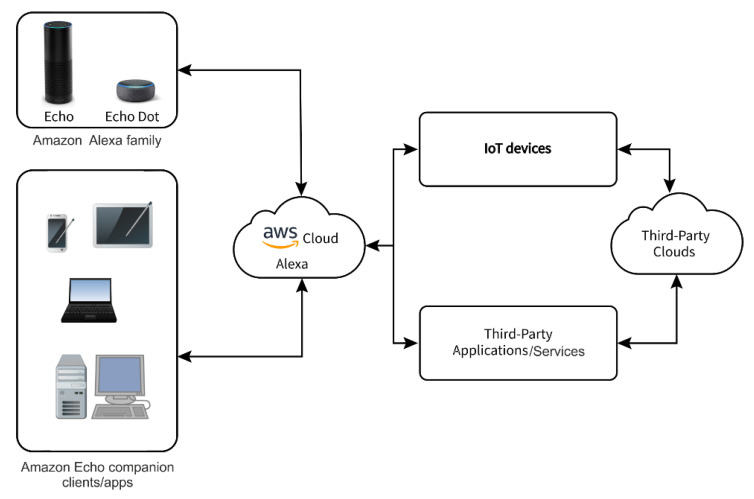
Amazon Alexa ecosystem.

The architecture of smart-home personal assistant systems for communication with IoT devices is designed in such a way that communication does not take place directly with IoT devices, but requests to go through the cloud. An example of a smart-home personal assistant architecture is shown for the Amazon Alexa ecosystem in Figure 8. A smart speaker device for voice control such as Amazon Echo Dot is required to record a voice request. The key component is the cloud, in this case the Amazon cloud platform, which includes Alexa Voice Service (AVS) as well as other cloud services such as authentication, data management, and logging. Access to cloud services requires a personal device to run companion Alexa applications, such as the Amazon Alexa application for Fire OS, iOS, or Android, referred to as companion clients. Users can also use web browsers to access the cloud. Alexa ecosystem can be extended by connecting compatible IoT devices, their cloud services and third-party applications for various other services [35]. This approach has an impact on the reaction to a possible DDoS attack on the smart home environment as well as on the resistance of IoT sensors to DDOS attacks.

The case study did not use the voice recognition of these devices, but the control of the respective IoT sensors via mobile applications smart-home personal assistants.

#### 2.4.1. Athom Homey

**Figure 9:**
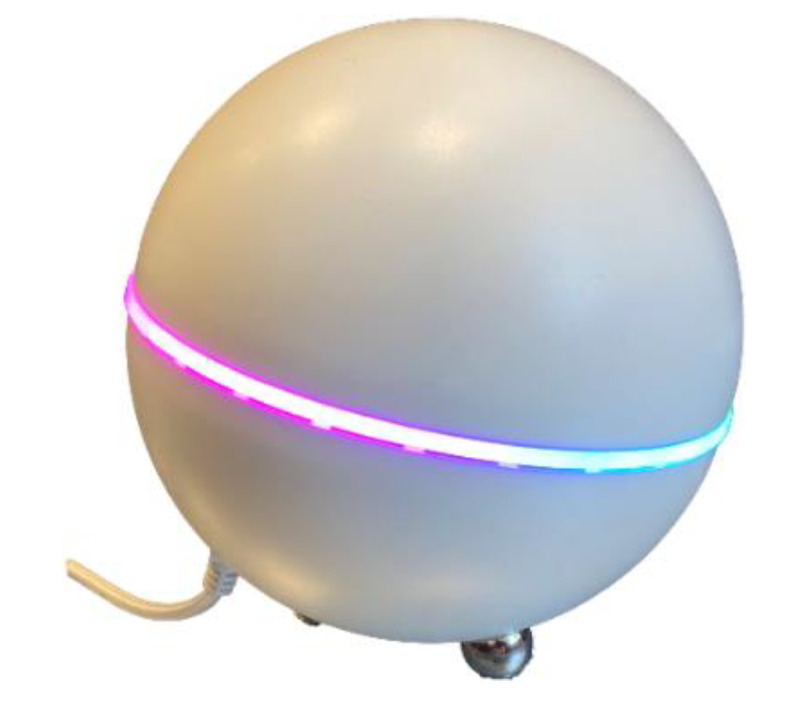
Smart-home controller Athom Homey Pro 2.0.

Homey is a smart-home voice-controlled device created by Netherlands-based startup Athom, Figure 9. Homey supports a variety of communication techniques for home automation, including Wi-Fi, Infrared, Zigbee, Z-Wave, Bluetooth, and 433 MHz and can communicate with a bunch of differently configured gadgets at once. Homey is a voice-controlled device, i.e., it is possible to send voice commands directly to Homey and so to control devices added to Homey ecosystem.

It provides its own mobile application for managing smart devices in the home, as well as for displaying data from IoT sensors. The service consists of multiple wireless technologies enabling connections to wireless devices, and congregates these on a single platform which connects to the Internet. It uses local or cloud secure connection. Homey Cloud Backups automatically creates a backup of Homey every night [36].

Athom Homey Pro 2.0 was used for the experiments performed, and the control of connected Philips Hue bulb and Fibaro Wall Plug was handled by a Homey mobile app provided by the Athom.

#### 2.4.2. Amazon Echo

Amazon’s Alexa is an intelligent personal assistant developed for use with the Amazon Echo. The Amazon Echo family of smart devices with a microphone and speaker connects to the intelligent cloud-based voice service, Alexa Voice Service (AVS). With Alexa as a voice-activated personal assistant, Echo is able to do a variety of things, such as manage to-do lists, play music, set alarms, place orders, search for information, and control other intelligent devices. Another great aspect is that Echo can also control the third-party hardware and software attached to it. It can control IoT devices such as smart-bulbs or play music from several streaming services.

The user can also define the so-called Skills. Skills provide a new channel for new content and services. Skills can be triggered by specific voice commands and allow customers to use their voices to perform daily tasks such as checking messages, listening to music, playing games, and more.

The Echo device is set up either using the mobile application for iOS or Android or via the website https://alexa.amazon.com. Echo requires a connection to a local Wi-Fi network and requires a constant connection to the Amazon cloud, as all voice requests are processed in a cloud environment. Echo and companion apps do not communicate with each other, but both are connected to the Amazon cloud, which acts as a communication proxy.

The Amazon Alexa ecosystem makes it available for users to place multiple devices in their homes into a comprehensive smart home system. For management purposes, Amazon provides functions for creating different groups of devices, assigning devices to specific rooms, assigning Echo devices to connected devices, and more [37,38].

**Figure 10:**
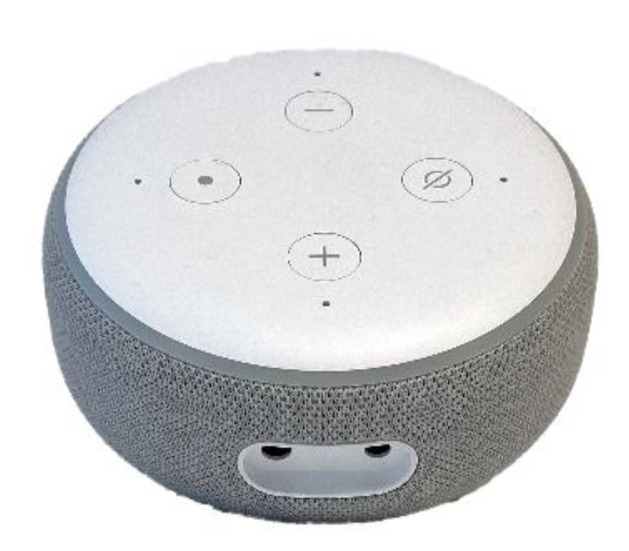
Echo Dot (3rd Gen)—Smart speaker with Alexa.

Amazon Echo Dot 3 was used for the experiments performed, Figure 10, and the control of connected Philips Hue bulb and Fibaro Wall Plug was handled by an Amazon Alexa mobile app provided by the AMZN Mobile LLC.

#### 2.4.3. Google Home

Google Assistant is a smart personal assistant developed for use with Google Home. Google Home as a smart home centering product was launched by Google Enterprise in 2016.

Google Home functionality consists primarily of the following features: (i) information retrieval—Google Home allows users to perform voice searches and life reminders; (ii) multimedia control—Google Home can act as a home wireless multimedia center with an integrated wireless speaker device; (iii) environmental control—Google Home allows to centrally control smart devices in a smart home system.

Among them, the main function is the voice assistance of Google Assistant, where the system responds based on the user’s voice requests. With Google Assistant and Google Home, the user can manage a large number of services and smart devices including sensors and actuators. Google Assistant is a service that uses artificial intelligence, machine learning, speech recognition, and collected data. The main processing is not performed on the device, but on the cloud [39,40]. Google Home is operational with wake word, “Ok Google” or “Hey Google.” Parallel to the Alexa Skills, in the Google environment there are the Google Actions.

Google Home is primarily a cloud-based service and requires a connection to a local Wi-Fi network. However, when the main router is offline or if it loses Wi-Fi connection, Google Home starts to broadcast a local Wi-Fi beacon for set and, consequently, the Google Home goes into set up mode where its own unsecured Wi-Fi is established.

**Figure 11:**
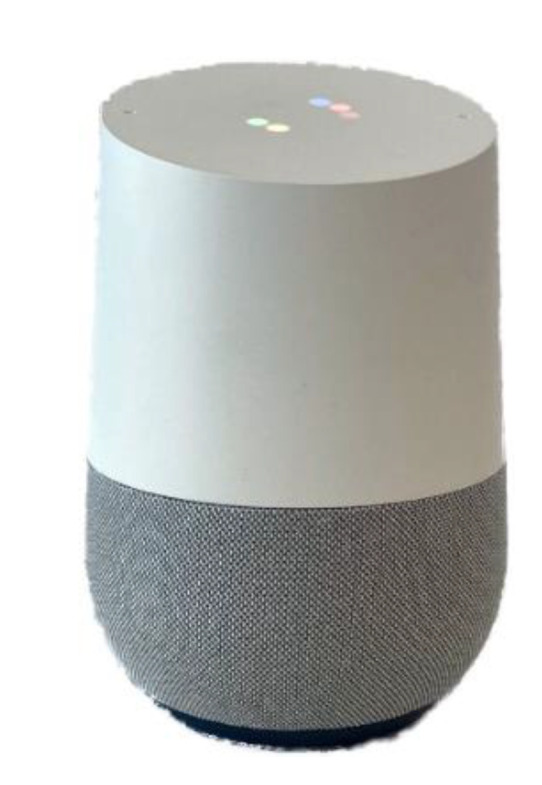
Google Home EU.

Google Home EU was used for the experiments performed, Figure 11, and the control of connected Philips Hue bulb and Fibaro Wall Plug was handled by a Home mobile app provided by the Google.

## 3. Results

This section describes the experimental scenarios used to test communication with IoT sensors during a DDoS attack as well as the results obtained. The test facility is based on the created Ansible real-time attack environment. Testbeds based on real systems, as opposed to simulation or emulation-based tools, provide realistic conditions, real platforms and applications, and have proven to be the best for network experiments [41]. Especially for DDoS attacks, and such an extremely complex ecosystem as a smart home environment, live testing is the best way to achieve realistic results. All attacks in the case study were conducted as controlled attacks under continuous supervision in an isolated research computer network.

**Figure 12:**
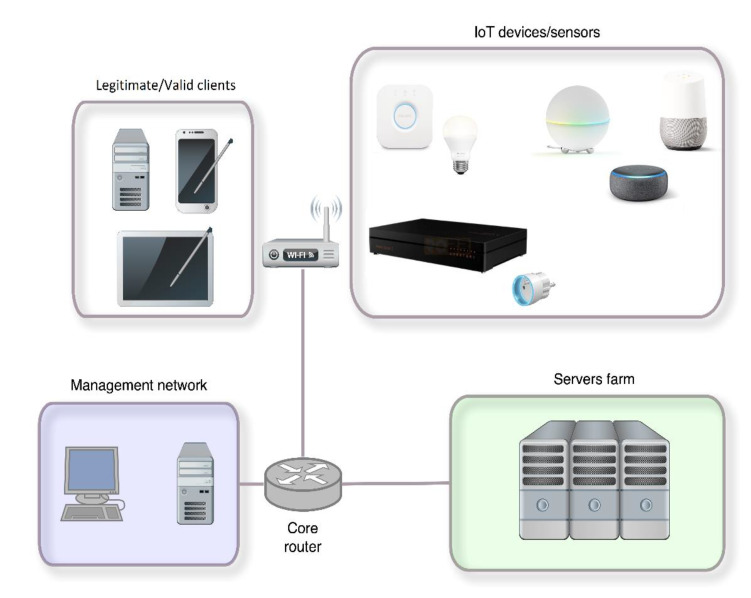
Structure of experimental environment.

The article examines how IoT devices and the entire smart home behave if they become victims of a DDoS attack aimed at the smart home from the outside. The question of security was asked in terms of whether a legitimate user could continue to control and receive information from the IoT sensors available during normal operation of the smart home, and the structure of the test environment was created accordingly, Figure 12. All devices used in the case study were connected to a central Wi-Fi router, which created a local network for the smart home. Test DDoS attacks came to this network from outside the Internet. The open ports of the selected device were attacked, which were detected during the first phase of the experiments by the scanning process on the devices using the nmap port scanner, where a full port scan was performed for each device tested.

For each of the combination of scenarios, both SYN flood attack and HTTP Get flood attack were performed, or SSL/TLS flood attack, if a port was open for it. The tests were performed in three different scenarios, each lasting 90 s on the same network topology. The authors’ previous experience with DDoS attacks [17,28,30] conducted on various devices, pointed to the fact that a medium-sized botnet, as used in the experiments in the article, can completely flood the device in 60 s, and the experiment will show the result. Another 30 s were examined to capture the recovery of the IoT device after the end of the 60 s attack.

The case study was done both as an attack on central units managing IoT sensors directly, as well as an attack on smart-home personal assistant systems, with which the user can control IoT sensors. During the attack, the possibility of communication of the user with the given IoT sensor was tested via the mobile application of the device manufacturer or via the mobile application of the smart-home controller or personal assistant.

The success of the attack was evaluated, i.e., whether communication with the IoT sensor was disabled or restricted. The congestion of the selected port was also checked by a valid client on each of the attacked ports, so that it was clear that the DDoS attack congested the given port.

### 3.1. Scenarios

DDoS attacks on the smart home environment took place for various variations of IoT devices, sensors, and mobile applications. Accordingly, the scenarios are divided into which devices were attacked and by which mobile application the functionality of the communication between the user and the respective IoT sensor was tested. It is necessary to point out that it is not possible to attack selected sensors communicating via ZigBee or Z-Wave protocol directly from the Internet in the form of DDoS attacks, but it is necessary to attack the gateway that communicates with them via the wireless protocol.

Each scenario consists of an attacking servers farm that performed a DDoS attack for 90 s; the response sensor tested; a legitimate user who tried to communicate with the sensor; an attacked target responsible for mediating communication with the sensor and the user; and a mobile application through which the user interacted with the sensor, Figure 12. The prototype of a smart house contains a set of objects and can be viewed as a multi-agent system.

#### 3.1.1. Scenario 1

The DDoS attack was performed directly on the gateway responsible for protocol communication with the respective sensor, while the user tries to communicate with the sensor using a mobile application issued by the manufacturer for the gateway. During the DDoS attack on the Philips Hue Brigde, which is responsible for communicating with the smart-bulb using the ZigBee protocol, the user’s effort was to turn the smart-bulb on and off throughout the attack using the Philips Hue mobile app. During the DDoS attack on Fibaro Home Center 3, which is responsible for communicating with the Fibaro Wall Plug using the Z-Wave protocol, the user’s attempt was to turn the Fibaro Wall Plug on and off using the Fibaro Home Center mobile app.

#### 3.1.2. Scenario 2

The DDoS attack was performed directly on the gateway responsible for protocol communication with the respective sensor, while the user tries to communicate with the sensor using smart-home controllers or personal assistant’s mobile applications. During the DDoS attacks on Philips Hue Brigde, which is responsible for communicating with the smart-bulb using the ZigBee protocol, the user’s effort was to turn on and off the smart-bulb throughout the attack using the Homey mobile app, Amazon Alexa mobile app, or Google Home mobile app. During the DDoS attack on Fibaro Home Center 3, which is responsible for communicating with the Fibaro Wall Plug using the Z-Wave protocol, the user’s attempt was to turn the Fibaro Wall Plug on and off using the Homey mobile app, the Amazon Alexa mobile app, or the Google Home mobile app.

#### 3.1.3. Scenario 3

The DDoS attack was performed directly on the devices of smart-home controllers or personal assistants, while the user tries to communicate with the sensor using the mobile applications of the respective smart-home controller or personal assistant. For DDoS attacks on the smart-home controller Athom Homey, the user used the Homey mobile app; when attacking a personal assistant device Amazon Echo Dot, the user used the Amazon Alexa mobile app; and when attacking a Google Home device, the user used the Google Home mobile app. Testing was performed for both the Philips Hue smart-bulb and the Fibaro Wall Plug.

#### 3.1.4. Arrangement of Attacks

The combination of examined options between the devices that were attacked and which mobile application tested the functionality of communication between the user and the respective IoT sensor is captured in Table 1. Testing in all cases was performed for both SYN flood attack and HTTP Get flood attack.

Moreover, a special case was tested. As mentioned in Section 2.4.3, when Google Home loses its connection to the Wi-Fi network, it will start creating its own unsecured Wi-Fi network with the same name as when connecting the Google Home device to the smart home environment. The aim is still to maintain the ability to communicate with the IoT devices it controls. However, this solution has its limits. As was found during testing, Google Home has a default network mask on this network set to /29, which limits the network range to only 8 addresses and 6 devices. During the testing, it was not possible for the first time to connect a smartphone with a mobile application for sensor control to this network, as all addresses were already occupied. Google Home had to be restarted for a successful connection. In addition, from the point of view of DDoS attacks, such a case is not relevant to other investigations, as in the event of a Wi-Fi network failure, it is not possible to carry out attacks from the Internet.

The Fibaro Home Center 3 has a similar function, when after a Wi-Fi network failure, the device creates its own Wi-Fi network and the user can manage it via the local web interface provided by the manufacturer based on the data provided on the back of the device. In this case, it is a secured Wi-Fi network.

### 3.2. Experimental Results

Prior to the individual DDoS attacks, a phase of scanning devices for open ports took place, through which a DDoS attack could be conducted. Table 2 lists the identified open ports that were used to attack the devices.

The effects of DDoS attacks on the communication between the user and the sensor are further divided according to the type of DDoS attack.

#### 3.2.1. SYN Flood Attack

The measured results for the SYN flood attack for all performed experiments listed in Table 1 showed unrestricted communication during the DDoS attack, i.e., the user could continuously control the sensor through the selected application. There were no outages or communication delays for any port for the devices or for any mobile application.

In addition, for scenarios 3, voice commands were also used during the attack for the devices of smart-home personal assistants Amazon Echo Dot and Google Home; both devices answered the questions asked fluently, the DDoS attack did not affect them.

#### 3.2.2. HTTP Get Flood Attack

The HTTP Get flood attack results were not the same for all devices, they varied for devices and also for scenarios. As shown in Table 3, some cases showed unrestricted communication between the user and the sensor, some responded with delays or outages, and in others the communication was completely inoperable.

- Cases with fully operational communication.

All cases identified in Table 3 as “Fully”, allowed for the user consistent communication with the sensor during a DDoS attack; the mobile application was fully controllable and there were no delayed reactions from the senators. Even in all scenarios 3, where personal assistant devices participated, the voice request to personal assistants was also tested during the attack, but they answered the questions asked smoothly by voice during the DDoS attack.

The following describes in more detail the course of attacks in cases where the communication was restricted or non-functional due to a DDoS attack.

- Cases with restrictions.
  1. Scenario 1; Philips Hue Bridge as attacked device; control by Philips Hue mobile app; with smart-bulb as testing sensor:

Immediately after the start of the DDoS attack, communication with the IoT sensor stopped working for 15 s; after 8 s even the mobile application declared that the connection is unavailable. After 15 s, however, the connection was re-established and operated without restriction until the end of the attack.

  
  2. Scenario 1; Fibaro Home Center 3 as attacked device; control by Fibaro Home Center mobile app; with wall plug as testing sensor:

Immediately after the start of the DDoS attack, communication with the IoT sensor stopped working. Only a few times was it possible to toggle the on/off state during the attack, but it did not work consistently. After the attack ended, the device made several on/off switches, even if the user no longer communicated.

- Cases with inoperable communication.
  1. Scenario 2; Philips Hue Bridge as attacked device; control by Homey mobile app; with smart-bulb as testing sensor:

Connected smart-bulb did not react at all during the whole attack. The mobile application occasionally reported a “time out after 30,000 ms” connection loss. At the end of the attack, the bulb switched the on/off state 20 times.

  
  2. Scenario 2; Fibaro Home Center 3 as attacked device; control by Amazon Alexa mobile app; with wall plug as testing sensor:

During the whole attack, the wall plug did not respond to user requests. Even the Amazon Alexa mobile app stated that it could not communicate with the sensor. On the other hand, it was possible to place a voice request to Amazon Alexa and Alexa responded fluently to the asked request even during the attack.

  
  3. Scenario 3; Athom Homey as attacked device; control by Homey mobile app; with smart-bulb as testing sensor:

Athom Homey could not be controlled using the mobile application. After 3 s, Homey even stopped communicating with Wi-Fi. Additionally, the connection to the application was lost. Communication did not return until the end of the attack, when the floods from the attacking computers stopped coming in and the smart-bulb subsequently switched the on/off state a few times, even though the user no longer communicated.

  
  4. Scenario 3; Athom Homey as attacked device; control by Homey mobile app; with wall plug as testing sensor

Athom Homey could not be controlled using the mobile application. After 3 s, Homey even stopped communicating with Wi-Fi. Additionally, the connection to the application was lost. Communication did not return until the end of the attack, when the floods from the attacking computers stopped coming in and the wall plug subsequently switched the on/off state a few times, even though the user no longer communicated.

## 4. Discussion

The effort of the performed experiments was to find out the behavior of the smart home environment from the point of view of the resistance of the user’s communication with the IoT sensor. The communication took place via a mobile application during a DDoS attack on the communication gateway.

The first collection of experiments for which the attacks were performed was the SYN flood attack. The results showed that for all variants of experiments performed for this type of attack, the attacks were ineffective and did not disrupt or restrict communication with the sensors. In addition, even a valid client that checked for port congestion during a SYN flood attack was able to establish a connection with that port. Given that SYN flood attack is one of the simplest and most common attacks seen on the Internet there are a number of defense mechanisms against it, e.g., SYN cookies [42]. Therefore, it can be expected that certain protection mechanisms are already implemented in IoT devices and so the devices are resistant to SYN flood attack.

During the execution of a SYN flood attack on the Amazon Echo device, it was not even possible to successfully repeat and confirm the attack mentioned in [20], where for a lower version of the Amazon Echo device it was achieved that the device had lost Internet connectivity.

HTTP Get flood attack, on the other hand, had a negative impact on several experiments performed. The situation is based on the fact that there are no simple defense mechanisms against HTTP GET flood attack and incoming requests are evaluated as legitimate traffic.

In both cases for Scenario 1, where the intermediary through which the sensor is connected to the Internet was attacked (Philips Hue Bridge, Fibaro Home Center 3) and where the user controlled the sensor via the mobile app of the manufacturer, communication with the sensor was restrictive and did not meet the requirements for continuous sensor control.

As can be seen from Table 3, in up to three out of four cases with inoperable communication, the smart-home controller Athom Homey was involved in the communication process.

On the other hand, the implementation of smart-home personal assistant devices into the communication process showed resistance to DDoS attacks. As mentioned in Section 2.4, smart-home personal assistant systems use cloud services for their activities, where communication leaves the smart-home environment and takes place via the cloud. Only in one of the tested cases, when a personal assistant was involved in the communication process, was the communication with the sensor was interrupted. Although both Athom and Fibaro use cloud services, they use them in a different way than Google and Amazon personal assistants. It turns out that the involvement of the cloud in the process of controlling the IoT sensor by users has its advantages, although the process of responding to user requests is slightly slower, the resilience of the system to flood attacks increases. To disrupt such communication, an attacker would have to have much more knowledge about how communication flows in a given smart-home environment and use more sophisticated tools to analyze the flow of information within the network, not just port scanning. The question of privacy remains open, as information about IoT devices and their use is also processed using a personal assistant cloud.

It was also interesting to note that the smart-home environment remembers part of the commands for sensors from the user, not all were lost during the DDoS attack, and after the attack, this part of the commands was executed immediately after each other in continuous sequence.

During device testing, it was found that when the Wi-Fi router in the smart-home environment fails, Google Home device tries to create its own Wi-Fi network to connect IoT devices. Such a network has significant limits on the number of devices connected. Although in this case it is not possible to perform attacks on the network from the Internet, the user also cannot control IoT sensors outside the house via the Internet. In addition, since it is an unsecured Wi-Fi network, it increases the risk of other attacks and an attacker could connect from outside the home and reconfigure the device or accessing information or settings.

In general, it can be stated that the performed experiments showed that a DDoS attack led from the outside on IoT devices in a smart home environment can be successful and have a significant impact on the communication and control of IoT sensors inside the smart home environment.

The recommendation for the smart-home environment for sensor control states that it is advisable to use a smart-home personal assistant system in addition to the application provided by the device manufacturer. From the point of view of resistance to DDoS attack, this system is usually more resistant to DDoS attacks and allows the user to control the IoT sensor even during a DDoS attack. The integration of a smart-home personal assistant system into a smart home environment not only solves the problem of technological fragmentation that arises when implementing an ecosystem of smart homes, but also increases the resistance of user communication with IoT sensors to DDoS attacks led from external networks.

## 5. Conclusions

This article describes a case study of a DDoS attack on IoT devices in a smart home environment and the impact of the attack on the communication and control of IoT sensors from the perspective of a user using smart home services. DDoS attacks were based on two types of attacks, namely SYN flood attack and HTTP Get flood attack, which flooded the IoT device with a number of packets in order to overwhelm the IoT device and thus prevent communication with IoT sensors. Three scenarios were used to test the attacks. The first is a DDoS attack on the sensor gateway, and the mobile application of the gateway manufacturer was used in the user’s communication with the sensors. The second scenario is a DDoS attack on the sensor gateway and the mobile application of the smart-home personal assistant system was used in the user’s communication with the sensors. The third scenario tested the DDoS attack directly on the smart-home personal assistant system and used the mobile application of the smart-home personal assistant system to communicate with the sensors. The experimental results showed different responses of the smart home environment when communicating with IoT sensors during the DDoS attack. The method of communication with the user IoT sensors through application of smart-home personal assistant system has proven to be the safest way to control IoT sensor.

The performed pilot testing can serve as fundamental research for further studies and it can be extended in the future by investigation of other IoT protocol behavior under various DDoS attack in such real world scenarios. Furthermore, the case of cloud communication between IoT sensors and personal assistant systems should also be studied in more detail and a strong framework resistant to DDoS attacks in the smart home can be proposed.

## Figures and Tables

**Table 1:** A brief overview of the cases used during the experimental testing.

Scenario	Attacked Device	Controlling Mobile App	Testing Sensor	Wireless Protocol
Scenario 1	Philips Hue Bridge	Philips Hue app	smart-bulb	ZigBee
Scenario 1	Fibaro Home Center 3	Fibaro Home Center app	wall plug	Z-Wave
Scenario 2	Philips Hue Bridge	Homey app	smart-bulb	ZigBee
Scenario 2	Philips Hue Bridge	Amazon Alexa app	smart-bulb	ZigBee
Scenario 2	Philips Hue Bridge	Google Home app	smart-bulb	ZigBee
Scenario 2	Fibaro Home Center 3	Homey app	wall plug	Z-Wave
Scenario 2	Fibaro Home Center 3	Amazon Alexa app	wall plug	Z-Wave
Scenario 2	Fibaro Home Center 3	Google Home app	wall plug	Z-Wave
Scenario 3	Athom Homey	Homey app	smart-bulb	ZigBee
Scenario 3	Amazon Echo Dot	Amazon Alexa app	smart-bulb	ZigBee
Scenario 3	Google Home	Google Home app	smart-bulb	ZigBee
Scenario 3	Athom Homey	Homey app	wall plug	Z-Wave
Scenario 3	Amazon Echo Dot	Amazon Alexa app	wall plug	Z-Wave
Scenario 3	Google Home	Google Home app	wall plug	Z-Wave

**Table 2:** Destination ports for DDoS attack.

Attacked Device	Attacked Open Ports
Philips Hue Bridge	80, 443, 8080
Fibaro Home Center 3	80, 443
Athom Homey	80, 443
Amazon Echo Dot	1080, 8888
Google Home	8008, 8443

**Table 3:** A brief overview of the scenarios used during the experimental testing.

Scenario	Attacked Device	Controlling Mobile App	Testing Sensor	Communication
Scenario 1	Philips Hue Bridge	Philips Hue app	smart-bulb	With restrictions
Scenario 1	Fibaro Home Center 3	Fibaro Home Center app	wall plug	With restrictions
Scenario 2	Philips Hue Bridge	Homey app	smart-bulb	Inoperable
Scenario 2	Philips Hue Bridge	Amazon Alexa app	smart-bulb	Fully
Scenario 2	Philips Hue Bridge	Google Home app	smart-bulb	Fully
Scenario 2	Fibaro Home Center 3	Homey app	wall plug	Fully
Scenario 2	Fibaro Home Center 3	Amazon Alexa app	wall plug	Inoperable
Scenario 2	Fibaro Home Center 3	Google Home app	wall plug	Fully
Scenario 3	Athom Homey	Homey app	smart-bulb	Inoperable
Scenario 3	Amazon Echo Dot	Amazon Alexa app	smart-bulb	Fully
Scenario 3	Google Home	Google Home app	smart-bulb	Fully
Scenario 3	Athom Homey	Homey app	wall plug	Inoperable
Scenario 3	Amazon Echo Dot	Amazon Alexa app	wall plug	Fully
Scenario 3	Google Home	Google Home app	wall plug	Fully
